# Long-Term Clinical Outcomes of Percutaneous Coronary Intervention in Saphenous Vein Grafts in a Low to Middle-Income Country

**DOI:** 10.7759/cureus.11496

**Published:** 2020-11-16

**Authors:** Ghufran Adnan, Intisar Ahmed, Javed Tai, Maria Ali Khan, Hammad Hasan

**Affiliations:** 1 Cardiology, Aga Khan University Hospital, Karachi, PAK; 2 Biostatistics and Epidemiology, Aga Khan University Hospital, Karachi, PAK; 3 Cardiology, Queen Alexandra Hospital, Portsmouth, GBR

**Keywords:** percutaneous intervention, low to middle income countries, saphenous vein graft

## Abstract

Background

Revascularization of saphenous vein grafts (SVGs) is challenging and debated for the last few decades. The percutaneous revascularization of SVGs was reported to have poorer long-term outcomes than native coronary artery revascularization.

Purpose

We aim to study the peri-procedural complications and long-term outcomes of the percutaneous revascularization of SVGs in a low-middle-income country.

Methods

In this retrospective study, we included 110 patients who underwent percutaneous revascularization from January 2011 to March 2020 and followed them retrospectively for long-term outcomes and major adverse cardiovascular events.

Results

The mean age was 71 ±9, and 81% were male. The most common reason for the presentation was non-ST segment elevation myocardial infarction (NSTEMI) (46%). The mean follow-up period of the study was 48±27 months. The most common comorbidity was hypertension (86%). A drug-eluting stent (80%) was placed in most of the patients, followed by a bare-metal stent (BMS) (14%) and percutaneous balloon angioplasty (POBA) (6%). We did not find any significant difference in major adverse cardiac events (MACE) (P=0.48), target vessel revascularization (TVR) (p=0.69), and target lesion revascularization (TLR) (p=0.54) with drug-eluting stent (DES) as compared to either BMS or POBA. The mean period from coronary artery bypass grafting (CABG) to SVG percutaneous coronary intervention (PCI) was 15± 5.5 years. Multivariate Cox regression analysis showed that an acute coronary syndrome (ACS) event, stroke, and female sex were independently associated with MACE.

Conclusion

The long-term outcomes of SVG PCI are not affected by the types of stents. Female gender, ACS, and stroke are the independent predictors of MACE after SVG PCI, and statin therapy has a positive impact on the long-term outcomes of SVG PCI.

## Introduction

Cardiovascular diseases are the leading cause of death worldwide, and coronary artery disease accounts for more than 40% of cardiovascular deaths [1]. Coronary artery bypass grafting (CABG) is a recommended revascularization procedure for complex coronary artery disease associated with better long-term outcomes, especially in patients with left main or multivessel coronary artery disease [2].

Although CABGs overall are reported to have a lower rate of repeat revascularization than percutaneous coronary intervention (PCI), saphenous vein grafts (SVGs) are prone to early atherosclerosis and degeneration, leading to graft occlusion [3] As high as 20% of the SVGs develop obstructive lesions and occluded in the first 18 months due to accelerated intimal hyperplasia [4-5]. At 10 years, more than 50% of the SVGs were found to be occluded [6-7].

The revascularization of SVGs is challenging and has been a matter of debate for the last few decades. A re-do coronary bypass graft is not recommended in the setting of a patent internal mammary graft to the left anterior descending artery, as it is associated with high perioperative complications [8]. The percutaneous revascularization of SVGs was reported to have a lower success rate and poor long-term outcomes as compared to native coronary artery percutaneous revascularization [9-10].

SVG intervention is not similar to that of native coronary artery percutaneous coronary intervention (PCI). Many factors described in the literature make SVG intervention challenging. These may include the arterialization of the vein graft due to high pressure, leading to intimal hyperplasia and atherosclerosis, a thin cap of atherosclerotic plaque, which makes it vulnerable for distal embolization intervention, and a large thrombus burden, especially in acute occlusion [11-12]. All these factors, along with the release of neurohumoral factors, lead to no-reflow, which increases the risk of peri-procedure myocardial infarction and affects short and long-term outcomes [13].

As the prevalence of coronary artery disease increases in low-middle-income countries, it is essential to generate local evidence to increase the effectiveness of coronary artery disease treatment strategies. We aim to study the peri-procedural complications, as well as the long and short-term outcomes of SVG PCI in a low-middle-income country.

## Materials and methods

We conducted a retrospective study at a major tertiary care hospital in Karachi. We enrolled in a cohort of patients from the last 10 years, i.e., 2010-2019, aged >18 years old who underwent PCI of SVG. Data were collected by reviewing the medical records of patients who fulfill inclusion criteria 1) >18 years of age, 2) PCI of the saphenous vein graft with either balloon angioplasty or stent (drug-eluting stent (DES) and bare-metal stent (BMS)). We excluded the patients who underwent PCI of native arteries.

We assessed demographic parameters, comorbidities at presentation, family history, cardiovascular risk factors, medications, previous PCI, and ejection fraction from the database. Procedural features were also recorded, i.e., native vessel disease, graft vessel disease, PCI of graft, type of procedure, reference vessel, number of stents, stent length, stent diameter, target vessel revascularization, guide catheter, guidewire, aspiration, thrombus, pre-dilation, post-dilation, and percentage of stenosis. We also followed for post-procedure events.

Operational definition

Event: Major adverse cardiac event (MACE) defined as cardiac death, myocardial infarction, stroke, and revascularization (including both target vessel revascularization and target lesion revascularization).

Target vessel revascularization (TVR): Defined as repeat percutaneous intervention of previously stented SVG graft.

Target lesion revascularization (TLR): Defined as repeat percutaneous intervention within 5 mm proximal or distal of previously stented SVG graft.

Statistical analysis

Data were analyzed using statistical software STATA (version 14.2; StataCorp, College Station, TX). In the descriptive analysis, mean and standard deviation were calculated for quantitative variables and proportions for qualitative variables, including the dependent variable. We did a univariate analysis with Cox proportional hazard regression in which association between independent variables was observed with the outcome. The final multivariable Cox regression model was developed using a stepwise approach through model building, considering p-value <0.05 statistically significant. After that, the global test for the proportional hazard assumption was performed. Acute coronary syndrome (ACS) event, stroke, and sex remained in the parsimonious model. Survival analysis was demonstrated by Kaplan Meier curves and compared with the log-rank test.

## Results

Between January 2011 and March 2020, a bypass graft angiogram was performed in 650 patients, out of which 111 patients with PCI of SVG were included. The mean age was 71 ±9, and 81% were male. The most common reason for the presentation was non-ST segment elevation myocardial infarction (NSTEMI) (46%). The mean follow-up period of the study was 48±27 months. The baseline characteristics of the patient are shown in Table 1. The most common comorbidity was hypertension (86%), followed by diabetes (66%), dyslipidemia (24%), stroke (3.60%), and chronic kidney disease (CKD) (15%).

**Table 1:** Baseline clinical characteristics of patients PCI: percutaneous coronary intervention; NSTEMI: non-ST segment elevation myocardial infarction; STEMI: ST-segment elevation myocardial infarction; ACE: angiotensin-converting enzyme

Age	71 ±9
Gender (male)	90(81%)
Hypertension	96(86%)
Diabetes	73 (66%)
Chronic kidney disease	17(15%)
Dyslipidemia	27(24%)
Stroke	4(3.6%)
Left ventricle ejection fraction<45%	57(51%)
Previous PCI	23(21%)
NSTEMI	51(46%)
STEMI	11(10%)
Unstable angina	23(21%)
Stable angina	26(23%)

Beta-blocker	97(87%)
Nitrate	27(24%)
ACE inhibitors	35(31%)
Calcium channel blocker	32(28%)
Statin	109(98%)
Angiotensin receptor blocker	24(21%)

The patients' angiographic and procedural characteristics of SVG PCI are shown in Table 2.

**Table 2:** Patients' procedural characteristics LM: left main; LAD: left anterior descending artery; LCX: left circumflex artery; RCA: right coronary artery; LIMA: left internal mammary artery; SVG: saphenous vein graft; OM: obtuse marginal artery

Native LM Disease	38(34%)
Native LAD disease	108(97%)
Native Ramus disease	11(10%)
Native LCX disease	104(94%)
Native RCA disease	106(96%)
LIMA-LAD disease	14(13%)
SVG-LAD disease	15(14%)
SVG-Diagonal disease	29(26%)
SVG-Ramus disease	8(7%)
SVG-OM disease	74(67%)
SVG-RCA disease	68(61%)
Thrombus	21(19%)
Aspiration	24(22%)
Stenosis percentage>90%	84(75%)
Guide catheter	
Left coronary bypass catheter	52(47%)
Judkins right	28(25%)
Amplatz left	5(4%)
Multipurpose catheter	25(23%)
Internal mammary catheter	1(1 %)
Guidewire	
Balance middleweight (BMW) wire	85(77%)
Run-through	11(10%)
Whisper	3(2.70%)
Cougar XT	3(3%)
Miracle	2(2%)
Fielder FC	1(1%)
Sion	6(5%)
Dilation	
Pre-dilation	59(53%)
Post-dilation	75(67%)
Maximum post-dilation	18±4.6
Stent size	
Stent diameter	3.23±0.5
Stent length	22.3±9
Stent number	
1	76(68%)
2	23(21%)
3	6(5%)
Stent type	
Drug-eluting stent (DES)	89(80%)
Bare metal stent (BMS)	15(14%)
Percutaneous balloon angioplasty (POBA)	7(6%)

Most patients in our study who had SVG PCI were also found to have significant (>50% for left main, >70% for other vessels) disease in native vessels. The most common disease vessels were SVG to OM and SVG to RCA grafts. We observed that the most common graft in which PCI was performed was SVG to OM (52%). However, patients with a critical disease in a left internal mammary artery (LIMA) graft were less as compared with an SVG graft, yet adverse events significantly occur more in patients with LIMA graft disease. Multivessel graft PCI was performed in 6% of patients. Most of the grafts engaged with LCB (47%) and BMW wires (77%) were able to cross the lesion successfully. A drug-eluting stent (80%) was placed in most of the patients, followed by BMS (14%) and POBA (6%). We did not find any significant difference in MACE, TVR, and TLR with DES as compared to either BMS or POBA. Nevertheless, the higher the number of stents in a patient, the more the likelihood of adverse outcomes. The average stent diameter 3.23±0.5, and length were 22.3±9. Direct stenting was done in 41% of patients. It was observed in patients in whom aspiration thrombectomy was performed either for large thrombus burden or no re-flow associated with adverse outcomes. It noted that patients with a high thrombus burden have adverse outcomes.

The mean period from CABG to SVG PCI was 15±5.5 years. However, it is noted that significant adverse events occur after five years of SVG PCI (p=0.08). The results of the events are shown in Table 3.

**Table 3:** Incidence of events TVR: target vessel revascularization; TLR: target lesion revascularization

MACE	18(16%)
TVR	10(9%)
TLR	5(5%)
Death	9(8%)
Cardiac death	3(3%)

**Figure 1:**
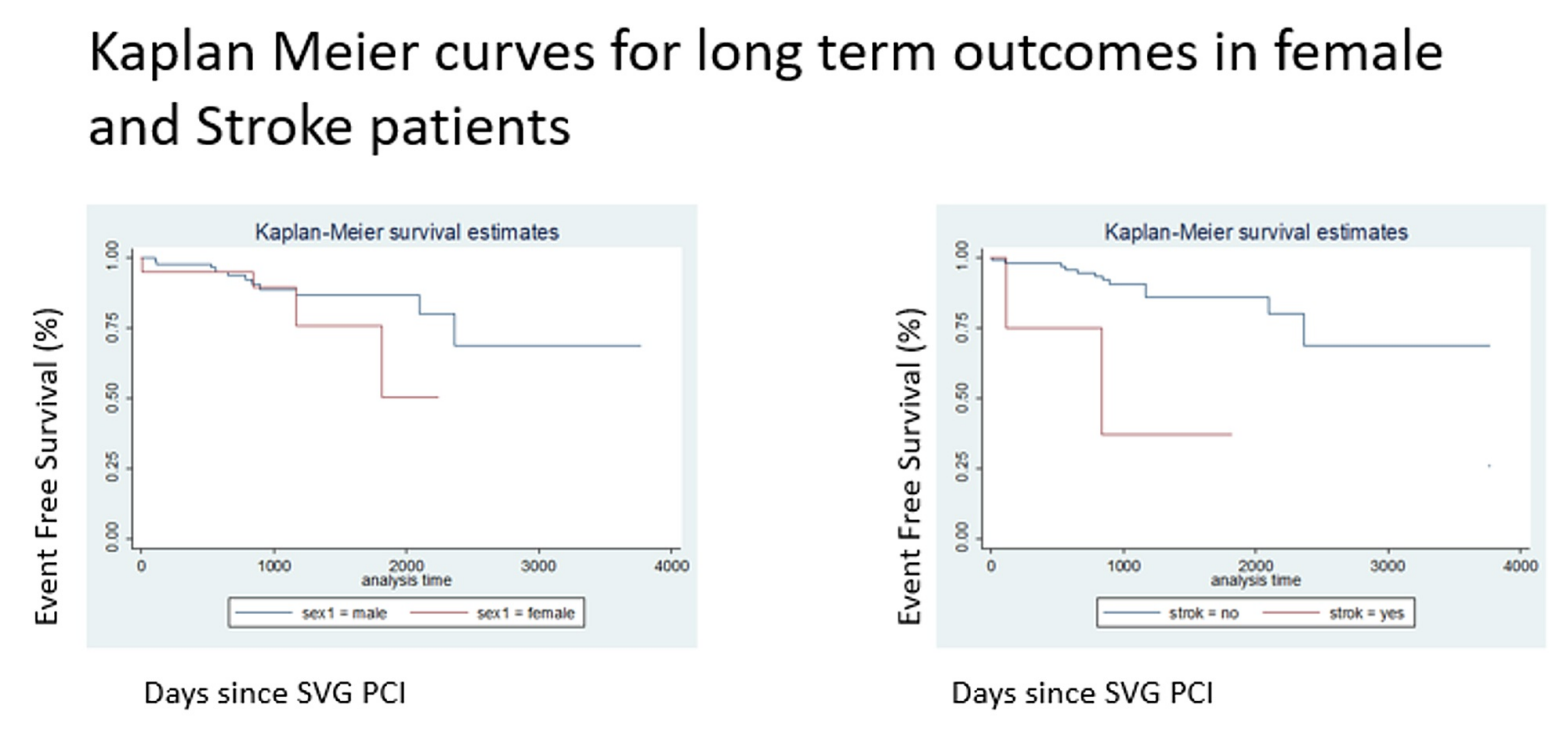
Kaplan Meier survival in females and stroke patients

The incidence of all-cause death was nine (8%), MACE 18 (16%), target vessel revascularization 10 (9%), and target lesion revascularization 5 (5%). Multivariate Cox regression analysis showed that an ACS event, stroke, and female sex were independently associated with MACE. Kaplan Meier survival (Figure 1) showed decreased survival in females, stroke, and acute coronary syndrome (Table 4).

**Table 4:** Predictors of MACE ACS: acute coronary syndrome; MACE: major adverse cardiac event

Predictors	HR	p-value	95% CI
Female sex	3.79	0.02	1.16-12.4
Stroke	9.79	<0.001	2.37-40.4
ACS	36.8	<0.001	7.6-178.2

## Discussion

It is one of the few studies from low and middle-income countries (LMICs) on the long-term outcomes of SVG PCI. Even in the contemporary era of advanced intervention techniques and embolic protection devices, studies from high-income and upper-middle-income countries have reported high peri-procedural complications and lower long-term outcomes of SVG PCI. In a meta-analysis by Sidik N et al., the peri-procedural complications of SVG-PCI were reported as 16%, and MACE was as high as 45% at the 36 months follow-up [14]. According to the ARRIVE (use of aspirin to reduce the risk of initial vascular events in patients at moderate risk of cardiovascular disease) study, patients undergoing SVG PCI with drug-eluted stents had higher two-year mortality, myocardial infarction, and stent thrombosis as compared to those who had native vessel PCI [15]. In another study by Eid-Lidt et al., SVG PCI was associated with high peri-procedural complications and lower MACE-free long-term survival [16]. The study reveals that most participants were males, and our study population was relatively older, with a mean age of more than 70 years. In a study by Redfors, the mean age of the participants undergoing saphenous vein graft PCI was 69.3 years, and 84% were males [17]. Although the male gender is an independent risk factor for coronary artery disease, females were reported to have poor outcomes after coronary intervention due to some reasons [18].

Ahmed et al. studied more than 1,000 patients with SVG grafts and found that female participants were older than males with a mean age of 69 years. Female gender was associated with poor outcomes after the percutaneous intervention [19]. On the other hand, the post-CABG trial showed that the male gender was inclined to have poor results than the female gender [20]. We found that the female gender was independently associated with significant MACE.

More than 85% of our study population had hypertension, and two-thirds of them had diabetes mellitus. Around 74% of the patients in our study presented with an ACS, which itself is an independent predictor of poor outcomes irrespective of intervention. Redfors et al. reported hypertension and diabetes in 91% and 44% of the study population, respectively, and only 56% of their patients presented with ACS [17]. In a study from China, 61% of the patients undergoing graft PCI were hypertensive, and 73% of the study population presented with ACS [21].

The mean time from CABG to the requirement of SCG PCI in our study population was around 15 years as compared with the other studies [14]. The majority of the patients underwent DES in our study, followed by BMS and POBA, respectively. Although SVG PCI with DES reported being superior to BMS, in terms of long-term outcomes, we did not find a statistically significant difference in the long-term outcomes and MACE among these intervention groups [15,22]. Stent diameter, length of the stent, and pre and post-dilatation were not found to be associated with MACE in our study population. A study by Leborgne supports direct stenting as pre-dilation is related to distal embolization of debris and increases peri-procedure myocardial infarction and increased CK-MB levels [23]. However, a retrospective study did not significantly differ in patients undergoing direct stenting compared to those who underwent pre-dilation in distal embolic protection devices [24].

Enormous thrombus burden and aspiration thrombectomy was associated with adverse outcomes. Enormous thrombus burden, especially in the setting of an ACS, is reported to have poor outcomes in patients undergoing SVG PCI [25]. Aspiration thrombectomy even during native coronary artery revascularization increases the risk of distal embolization and stroke [26]. However, embolic protection devices are recommended during SVG PCI to reduce incidents of distal embolization and no-reflow but those devices were not used in our study population due to limited availability [27].

Other than the female gender, ACS and stroke were independent predictors of MACE in our study population. In a pooled analysis of around 4,000 patients, Coolong et al. found that plaque burden large thrombus and SVG degeneration were the strongest predictors of MACE [28]. An ACS is caused by acute thrombotic occlusion of the SVG graft, which leads to distal embolization and no-reflow. Brodie BR et al. reported poor outcomes of SVG PCI in ST-elevation myocardial infarction due to distal embolization and no-reflow [29].

Our study has some limitations. As this is a retrospective, observational study and patients were not randomized, which may influence the results. The research was done in a single-center, and the sample size was small, which may not represent the whole population in our region. We do not have a comparison group, so it is difficult to establish the association of SVG PCI with mortality. We suggest prospective randomized studies to predict the long-term outcomes of SVG PCI in our region.

## Conclusions

Patients undergoing SVG PCI in our population are relatively older and predominantly male. The long-term outcomes of SVG PCI are not affected by the type of stents. Female gender, ACS, and stroke are independent predictors of MACE after SVG PCI.
